# Spooky action at a distance: neuromodulation, physiologic distress signals, and limb preservation

**DOI:** 10.1093/burnst/tkag017

**Published:** 2026-04-08

**Authors:** Ahmed Sami Raihane, Gabriela Morales Deusch, Charles Liu, Bijan Najafi, Wuquan Deng, Natasha G Dark, David G Armstrong

**Affiliations:** Division of Plastic & Reconstructive Surgery, Department of Surgery, University of New Mexico School of Medicine, 1 University of New Mexico, MSC10 5550, Albuquerque, NM 87131, United States; Pasadena City College, 1570 E Colorado Blvd, Pasadena, CA 91106, United States; University of Southern California Neurorestoration Center and Department of Neurological Surgery, Keck School of Medicine of the University of Southern California, 1200 N State St, Los Angeles, CA 90033, United States; Rancho Los Amigos National Rehabilitation Center, 7601 Imperial Hwy, Downey, CA 90242, United States; Department of Surgery, David Geffen School of Medicine at the University of California, Los Angeles, 10833 Le Conte Ave, Los Angeles, CA 90095, United States; Department of Endocrinology, School of Medicine, Chongqing Emergency Medical Center, Chongqing University Central Hospital, Chongqing University, No. 1 Jiankang Rd, Yuzhong District, Chongqing 400014, China; Division of Plastic & Reconstructive Surgery, Department of Surgery, University of New Mexico School of Medicine, 1 University of New Mexico, MSC10 5550, Albuquerque, NM 87131, United States; Department of Surgery, Keck School of Medicine of the University of Southern California, 1520 San Pablo St, Los Angeles, CA 90033, United States

**Keywords:** Diabetic foot ulcer, Chronic limb-threatening ischemia, Neuromodulation, Remote ischemic conditioning, Angiogenesis, Limb preservation

## Abstract

The rising global prevalence of chronic conditions, notably obesity and type 2 diabetes, demands innovative approaches to mitigate their health and economic impacts. Complications, including neuropathy and chronic limb-threatening ischemia (CLTI), dramatically increase the risk of lower limb amputation, cardiovascular events, and cerebrovascular events, underscoring the urgent need for effective interventions. Emerging neuromodulation and regenerative strategies provide novel approaches to addressing diabetes-related complications. High-frequency (10-kHz) spinal cord stimulation demonstrates marked pain relief and sensory improvement in patients with refractory painful diabetic neuropathy. Peripheral focused ultrasound, including splenic-targeted stimulation, shows promise in reducing systemic inflammation, accelerating wound repair, and enhancing vascular function. Remote ischemic conditioning leverages brief controlled ischemic reperfusion cycles to enhance microcirculation and promote diabetic foot ulcer (DFU) healing. In severe cases, surgical techniques such as tibial transverse transport and lateral tibial periosteum distraction stimulate angiogenesis and enhance distal limb perfusion. Integrated wound care protocols, incorporating these procedures alongside debridement, negative pressure wound therapy, and skin grafting, may further optimize outcomes. Collectively, these therapies address both local and systemic pathophysiology, frequently producing physiologic effects at sites distant from the primary intervention. These seemingly disparate therapies represent a single unifying concept: generating a physiologic effect at locations remote from the primary target. This systematic approach, engaging neural, vascular, and immune pathways, may be key to improving outcomes in DFUs and CLTI. Early clinical data appears promising; however, larger randomized trials are required to validate efficacy, refine patient selection, and determine optimal integration with standard care. If confirmed, these strategies may shift management toward patient-centered, regenerative interventions that preserve limbs, reduce recurrence, and enhance quality of life for the expanding global patient population. Further research is warranted to confirm or refute these early promising physiologic effects.

## Highlights

- Diabetic foot disease is fundamentally a systemic neurovascular and inflammatory disorder, not a purely local wound, explaining the high failure and recurrence rates of conventional limb-salvage strategies.
- Neuromodulatory and mechanotransductive interventions, including spinal cord stimulation, focused ultrasound, ischemic conditioning, and periosteal-based surgery, converge on a shared principle: inducing regenerative physiologic effects at sites remote from the point of intervention.
- Remote activation of neural, endothelial, and immune signaling pathways can enhance microcirculation, angiogenesis, and tissue resilience in ischemic limbs, reframing limb preservation as a biologically restorative rather than palliative goal.
- Early clinical signals across multiple modalities suggest improvements in pain, perfusion, wound healing, and limb salvage, but current evidence is limited by small cohorts, heterogeneous protocols, and incomplete long-term follow-up.
- Translation into routine practice will require rigorously designed multicenter trials with amputation-free survival, recurrence, and quality-of-life endpoints, alongside careful attention to cost, access, and procedural morbidity.

## Background

Emerging technologies, including neuromodulation and therapeutic ultrasound, are being actively developed to modulate metabolic and inflammatory function in chronic diseases such as obesity and type 2 diabetes. This is particularly important given the growing global burden of diabetes, which reduces life expectancy and imposes substantial healthcare costs [1]. Chronic hyperglycemia, endothelial dysfunction, and impaired angiogenic responses collectively contribute to peripheral vascular compromise, neuropathy, and poor wound healing, thereby increasing the risk of diabetic foot ulcers (DFUs) and chronic limb-threatening ischemia (CLTI) [2].

In the United States, an estimated 38.4 million individuals (11.6% of the population) have diagnosed diabetes, with an additional 8.7 million undiagnosed cases, and ~97.6 million adults are prediabetic, including almost half of those aged ≥65 years [1, 3]. Minority populations, including American Indian, Alaskan Native, and non-Hispanic Black adults, are disproportionately affected, reflecting persistent inequities in access to preventive care, vascular evaluation, and limb-salvage resources [3, 4]. These disparities are compounded by reduced access to specialized limb-preservation interventions, contributing to higher rates of adverse outcomes [5].

Globally, an estimated 800 million individuals live with diabetes, with nearly 75% residing in low- and middle-income countries [6]. This rise is driven by urbanization, aging populations, sedentary behavior, and increasing obesity prevalence, underscoring the need for scalable, non-pharmacologic, and cost-effective therapeutic strategies applicable across diverse healthcare settings [7].

Diabetes is associated with a wide spectrum of complications, including chronic kidney disease, retinopathy, neuropathy, cardiovascular disease, stroke, and peripheral vascular disease, as well as increased risks of infection, malignancy, and cognitive decline [8]. Of particular concern are DFUs and CLTI, which frequently coexist due to the synergistic effects of neuropathy and vascular insufficiency, creating a self-perpetuating cycle of ischemia, infection, and tissue loss [8, 9]. The global burden of peripheral artery disease and CLTI is expected to rise substantially over coming decades, driven by population aging and increasing diabetes prevalence [10, 11]. Despite advances in wound care and revascularization, DFUs remain associated with substantial amputation risk and low short-term healing rates in many real-world cohorts [12–17]. Recurrence after ulcer healing or limb-salvage intervention remains exceptionally common, exceeding recurrence rates observed in many aggressive cancers [18].

These outcomes underscore a fundamental limitation of conventional approaches that focus primarily on local wound management or macrovascular revascularization. Increasing attention has therefore shifted toward therapies capable of enhancing microcirculation, modulating systemic inflammation, and engaging neural and immune pathways that influence tissue repair beyond the immediate site of intervention [19]. This framework forms the conceptual foundation for the neuromodulatory, humoral, and mechanotransductive strategies reviewed in this article.

## Review

### Overview of neuromodulatory and regenerative strategies in diabetic foot disease

Recent advances in minimally invasive procedures, including spinal cord stimulation (SCS), peripheral focused ultrasound (pFUS), and remote ischemic conditioning (RIC), show promise in mitigating diabetes-related complications. Unlike conventional therapies that primarily target glycemic control or local wound treatment, these modalities directly influence neural, vascular, or immune pathways to improve tissue viability [20]. SCS is canonical electrical neuromodulation acting primarily via spinal and sympathetic pathways; by contrast, pFUS and RIC produce mixed neuromodulatory and humoral effects [21]. pFUS frequently acts through organ-reflex neural circuits that secondarily alter cytokine release, whereas RIC’s predominant effects are humoral and endothelial. Nitrite and nitric oxide (NO) signaling have been identified among the circulating mediators, and both therapies include a neural component as part of the cascade [22].

RIC is appealing because it is a low-cost, non-invasive modality, but human data are limited and trial results have been inconsistent across indications. Small randomized or proof-of-concept DFU studies have reported promising wound-healing signals; yet, sample sizes and follow-up durations are insufficient to confirm durable benefits [23]. Practical concerns include variable protocols, such as cycle duration and frequency, patient adherence, and possible unknown effects of repeated ischemia in patients with severe peripheral vascular disease [24].

**Figure 1:**
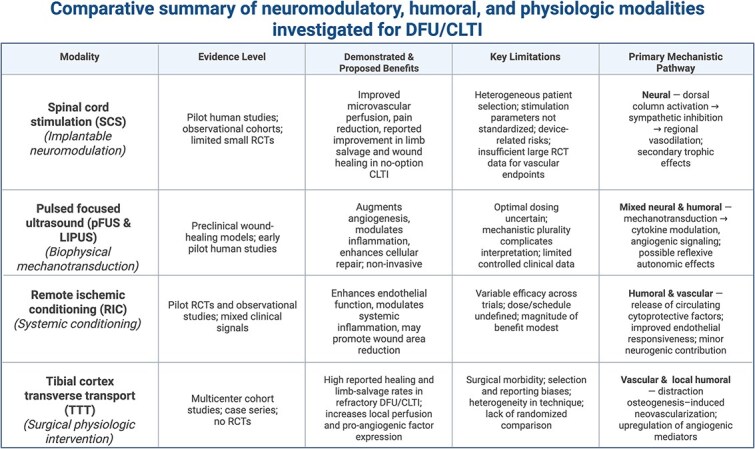
Comparative overview of neuromodulatory, humoral, and mechanotransductive interventions for diabetic foot ulcers and chronic limb-threatening ischemia (CLTI). Spinal cord stimulation (SCS), peripheral focused ultrasound (pFUS), low-intensity ultrasound stimulation (LIUS), remote ischemic conditioning (RIC), and tibial transverse transport (TTT) are organized by dominant mechanism of action, level of available evidence, representative clinical or preclinical findings, and key translational limitations. Although these modalities differ in proximal targets-neural modulation, humoral signaling, or biomechanical mechanotransduction, they converge on shared downstream effects including improved microcirculation, angiogenesis, endothelial function, and tissue repair. Evidence ranges from preclinical studies to early-phase human investigations and RCTs [23–29]. Created in BioRender. Neal, K. (2026) https://BioRender.com/eudkhz1

Techniques like tibial transverse transport (TTT) and lateral tibial periosteum distraction (LTPD) aim to enhance blood flow, promote angiogenesis, and accelerate wound healing, particularly in higher-grade DFUs. These approaches provide alternative strategies to conventional surgical and pharmacologic treatments and may help reduce the burden of severe diabetes-related complications [18, 25]. However, the current evidence base varies widely, with some modalities supported primarily by preclinical studies while others have early-phase human data or small randomized trials [26]. Larger multicenter randomized controlled trials (RCTs) remain necessary. A comparative summary of each therapy’s dominant mechanism of action, highest level of available evidence, representative findings, and major limitations affecting clinical translation is provided in Figure 1 [22–29].

Several non-invasive, non-pharmacologic therapies are emerging for chronic disease management, particularly due to their potential to reduce the substantial economic burden of these conditions. One such example is pFUS, a non-invasive neuromodulatory technique under active translational development that can engage organ-specific neuroimmune reflex circuitry, influencing inflammatory cytokine profiles and autonomic pathways involved in metabolic regulation [21, 30].

We also review the application of 10-kHz SCS via the Senza® system and its potential role in delivering neuromodulatory therapy for patients with chronic pain. SCS may improve microvascular perfusion through modulation of sympathetic tone, improved endothelial function, and changes in neuroimmune signaling, though mechanisms require further validation in diabetic populations [31, 32]. Additionally, we discuss Abbott Laboratories’ neuromodulation technology in the context of CLTI, including RCTs evaluating SCS implants as a therapeutic strategy to reduce pain and improve perfusion in ischemic tissues [33].

Furthermore, we examine peripheral focused ultrasound (pFUS) as a novel therapeutic approach for burn wound care, with potential relevance to diabetic patients and those with chronic limb ischemia. Preclinical and early translational studies demonstrate that targeted peripheral neuromodulation, including splenic-focused approaches, has been shown to modulate systemic inflammatory signaling and cytokine-specific neural pathways, providing mechanistic support for neuroimmune regulation rather than direct clinical outcome evidence [20, 29, 34]. While data appear promising, most findings remain early-phase, and translation to routine clinical care will require standardization of dosing parameters and target selection. RIC demonstrates potential as a non-invasive adjunct therapy for DFUs, complementing standard wound care without interfering with current treatment protocols. RIC has demonstrated mixed results in cardiac and vascular applications, and its role in DFU healing remains exploratory pending controlled clinical trials [35].

**Figure 2:**
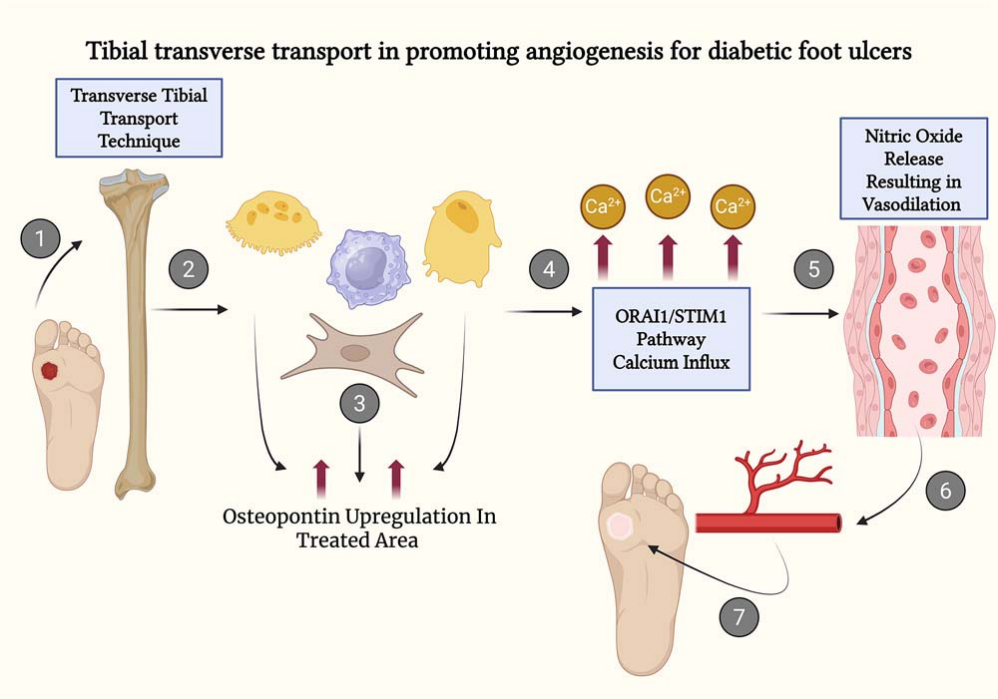
Proposed mechanistic cascade of tibial transverse transport (TTT) in diabetic wound healing. Mechanical distraction of the tibial cortex during TTT induces periosteal activation and upregulation of osteopontin (OPN). Increased OPN expression activates the Orai1/stromal interaction molecule 1 (STIM1) calcium entry pathway, resulting in intracellular calcium (Ca^2+^) influx and activation of eNOS. Subsequent nitric oxide (NO) release promotes vasodilation, enhanced microcirculation, angiogenesis, and distal ischemic tissue repair, supporting wound healing and limb preservation [36–38]. Created in BioRender. Raihane, S. (2026) https://BioRender.com/v61l054

Finally, we review TTT and integrated surgical wound treatment (ISWT) in severe diabetic foot cases (Figure 2). These interventions aim to improve limb reperfusion, with reported benefits including enhanced skin sensation, improved thermoregulation, and accelerated wound healing. Emerging evidence indicates that TTT upregulates osteopontin (OPN), activates the Orai1/STIM1 calcium signaling pathway, and increases NO production, thereby promoting angiogenesis in models of diabetic wounds [36].

Unlike pharmacologic or conventional surgical approaches, TTT functions as a regenerative mechanotransduction therapy, leveraging controlled biomechanical tension to stimulate angiogenic and microcirculatory responses [37]. While these mechanistic pathways have been primarily characterized in preclinical models, early clinical studies in patients with diabetic foot disease report improvements in distal perfusion and wound closure rates [38].

These technologies represent promising therapeutic strategies that minimize reliance on pharmacologic interventions, instead utilizing ultrasound-based approaches to modulate metabolic function and potentially normalize blood glucose levels in patients at risk for type 2 diabetes. Leveraging such innovations, neuromodulation may serve as a non-invasive treatment option for CLTI.

A less invasive surgical approach, periosteal distraction (PD), has been proposed to operate via a similar mechanism [39]. PD promotes bone regeneration by stimulating the periosteum, a highly vascular fibrous membrane covering bone surfaces that is crucial for bone growth and repair. In this technique, the periosteum is carefully elevated from the underlying bone, creating a potential space that enhances vascularization and stimulates mesenchymal stem cell (MSC) proliferation and differentiation into osteoblasts. This process activates key regenerative signaling pathways, including bone morphogenetic protein (BMP) and Wnt pathways, which are critical for tissue repair and angiogenesis [40, 41]. To date, most evidence from PD’s regenerative effects is derived from animal models, though its lower surgical complexity makes it an excellent candidate for clinical translation [42]. Compared to TTT, PD may reduce procedural morbidity and eliminate the need for external fixation, but head-to-head human studies are sparse [43]. Continued investigation is needed to determine the wound severity threshold for each technique, alongside the optimal patient population [43].

For conceptual clarity, this review distinguishes two mechanistically distinct but potentially convergent categories. Direct neural modulations, exemplified by SCS, refers to interventions that apply electrical energy to alter afferent/efferent neural signaling and autonomic outflow. High-frequency SCS has been studied in randomized clinical trials for painful diabetic neuropathy (PDN) and represents a form of electrical neuromodulation [28]. Conditioning therapies, including RIC, pFUS, TTT, and LTPD, primarily act by inducing circulating mediators, endothelial programs, or mechanically mediated angiogenic responses that secondarily influence immune and neural tone. While these two categories share vascular and inflammatory effects relevant to wound healing, conflating their proximal mechanisms risks misleading translational inference [29, 44].

### Neuromodulation and related neuroimmune interventions for chronic pain: Insights from the Neuromodulation Appropriateness Consensus Committee of International Neuromodulation Society

The Neuromodulation Appropriateness Consensus Committee of the International Neuromodulation Society (INS) has spent years evaluating the safety and efficacy of neurostimulation for conditions such as chronic critical limb ischemia, generalized chronic pain, peripheral neuropathy, and angina, as well as its integration into clinical practice [45]. The INS defines neuromodulation as the targeted alteration of nerve activity through chemical agents or electrical stimulation to restore function and alleviate symptoms such as pain, seizures, and spasticity in vulnerable patients [46].

**Figure 3:**
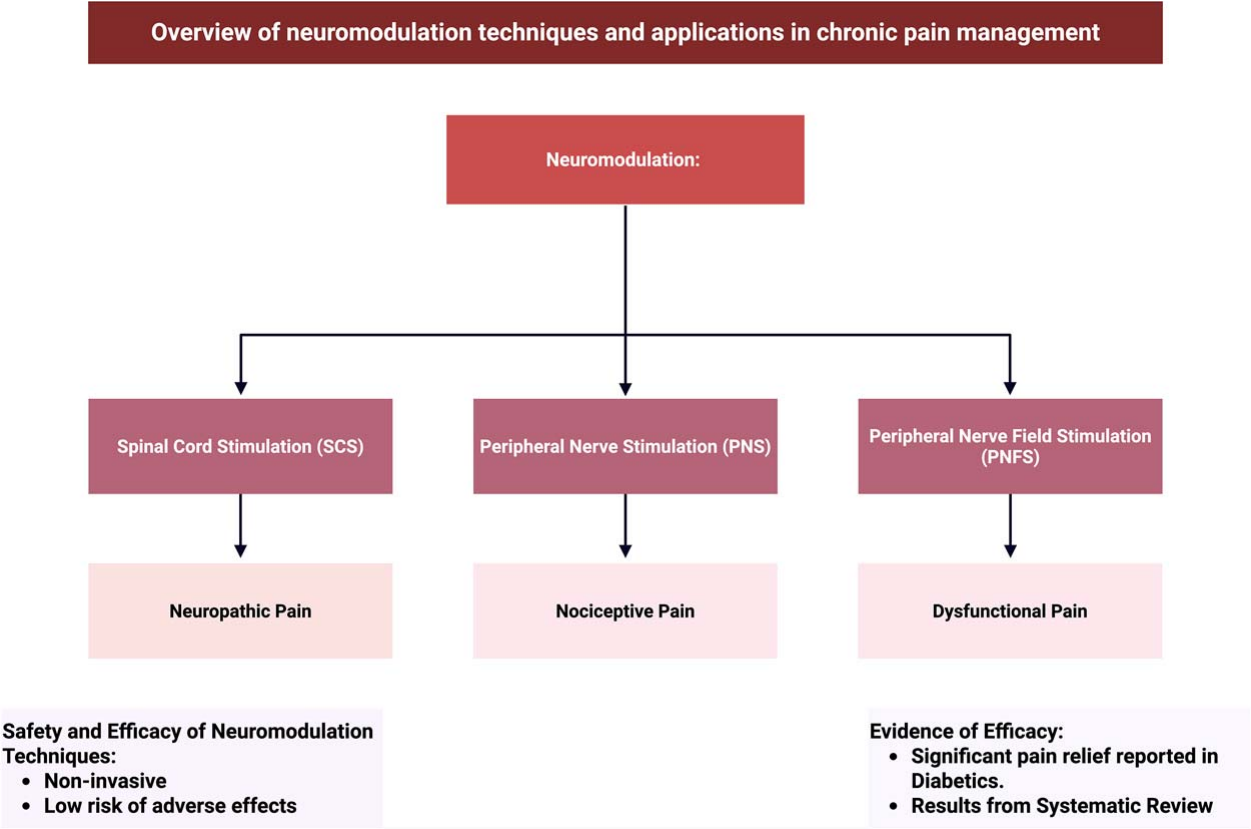
Overview of neuromodulation techniques and their clinical applications in chronic pain. Neuromodulation includes spinal cord stimulation (SCS), peripheral nerve stimulation (PNS), and peripheral nerve field stimulation (PNFS), each targeting distinct pain phenotypes. SCS primarily modulates dorsal column pathways and sympathetic tone for neuropathic pain, whereas PNS and PNFS may address nociceptive or mixed pain syndromes. These modalities are generally reversible and non-pharmacologic and may influence peripheral perfusion through autonomic and neuroimmune mechanisms. Created in BioRender. Neal, K. (2026) https://BioRender.com/mkd6u2r

Pain manifests in various mechanistic categories: neuropathic, nociceptive, mixed, and dysfunctional pain, and appreciating these distinctions is essential when selecting between SCS, peripheral nerve stimulation (PNS), and peripheral nerve field stimulation (PNFS) [47]. Pain types include neuropathic, nociceptive, mixed, and dysfunctional pain. In diabetic peripheral neuropathy (DPN), more than half of patients experience debilitating pain and significant reductions in quality of life, reinforcing the clinical urgency for modalities capable of modifying aberrant neural signaling when pharmacologic therapies lose efficacy or fail (Figure 3) [47].

**Figure 4:**
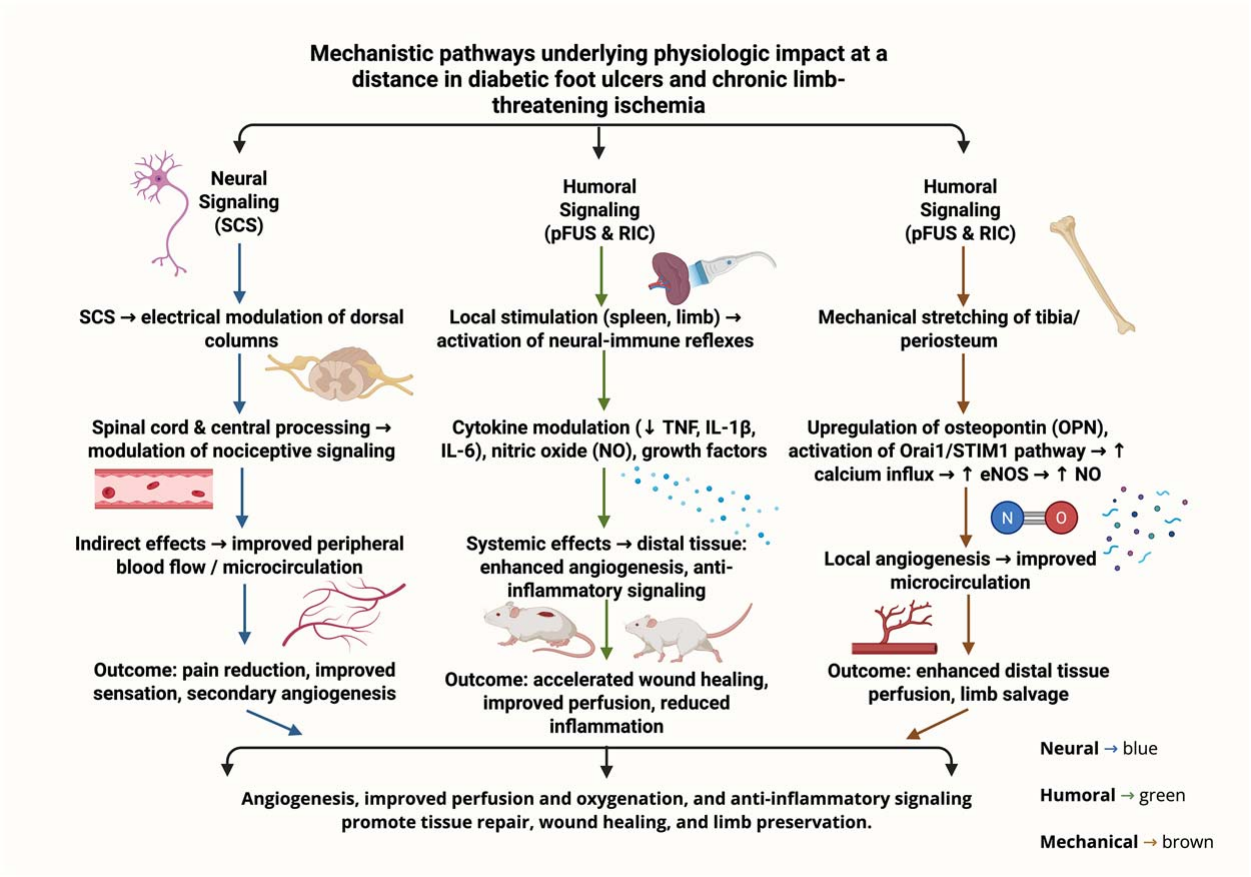
Mechanistic pathways underlying physiologic effects at a distance in diabetic foot ulcers and chronic limb-threatening ischemia (CLTI). Spinal cord stimulation (SCS) exerts predominantly neural effects via dorsal column modulation and sympathetic inhibition, indirectly improving peripheral blood flow and microcirculation. Remote ischemic conditioning (RIC) and peripheral focused ultrasound (pFUS) activate humoral and neuroimmune pathways, including modulation of tumor necrosis factor alpha (TNF-α), interleukin-1 beta (IL-1β), interleukin-6 (IL-6), and nitric oxide signaling. Surgical mechanotransduction, exemplified by tibial transverse transport, induces angiogenesis through periosteal activation, osteopontin upregulation, calcium signaling via the Orai1/STIM1 pathway, and eNOS activation. Despite differing proximal mechanisms, these therapies converge on enhanced microcirculation, angiogenesis, and distal tissue repair [20, 29, 44, 49]. Created in BioRender. Neal, K. (2026) https://BioRender.com/kw32apm

Although generally low risk, rare device-related complications have been reported [48]. A systematic review by Pluijims *et al.* evaluated SCS in painful diabetic polyneuropathy across three prospective case series and one retrospective cohort study (*n* = 25), demonstrating significant pain relief with no adverse events [49]. These findings support the potential of neuromodulation as a safe and effective therapeutic option warranting further investigation [49]. To situate these findings within the broader therapeutic landscape, we have included a comparative evidence figure (Figure 4) that synthesizes device mechanisms, clinical indications, safety profiles, and durability of benefit across SCS, pFUS, and RIC.

### Adjunct biophysical therapies in diabetic foot ulcer care

**Figure 5:**
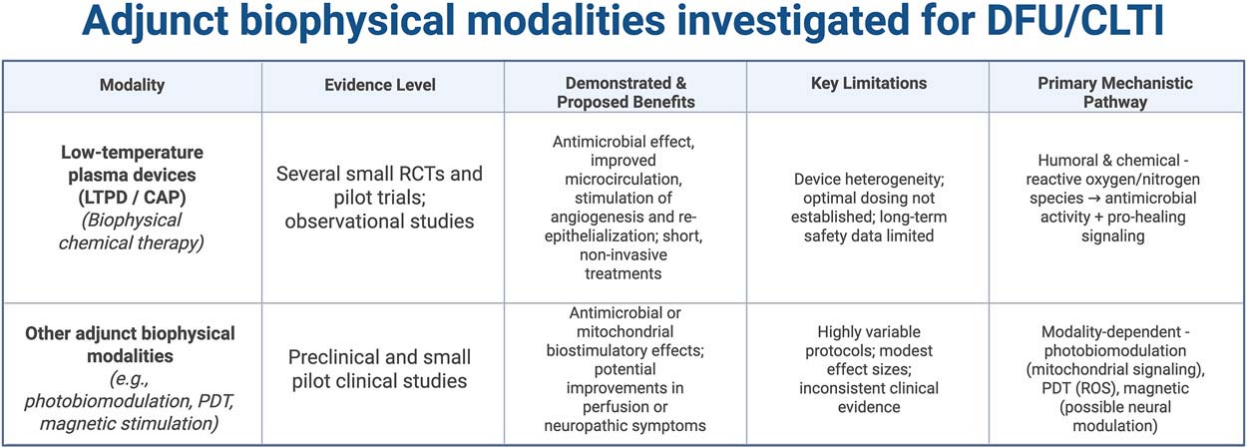
Adjunct biophysical therapies investigated for diabetic foot ulcers (DFUs) and chronic limb-threatening ischemia (CLTI). Adjunctive modalities include Cold atmospheric plasma (CAP), photobiomodulation therapy (PBMT), photodynamic therapy (PDT), and electromechanical stimulation such as ESWT. These therapies exert antimicrobial, mitochondrial, pro-angiogenic, or anti-inflammatory effects through modality-specific mechanisms, including reactive oxygen species (ROS) generation in PDT and mitochondrial signaling modulation in PBMT. Supporting evidence derives primarily from small RCTs, pilot clinical studies, and preclinical models, with heterogeneous protocols and limited long-term safety data. Created in BioRender. Neal, K. (2026) https://BioRender.com/nbu4u2q

In addition to neuromodulatory and surgical techniques, adjunct biophysical therapies have also shown early promise in DFU care (Figure 5). Photobiomodulation therapy (PBMT) (low-level laser therapy) has been studied in patients with neuroischemic DFUs, where it accelerated wound closure and improved neuropathic parameters [50]. Multiple PBMT studies with varied designs and parameters have reported healing improvements, although protocols such as wavelength and dosing varied widely [50]. Cold atmospheric plasma (CAP) therapy is yet another emerging adjunct: a meta-analysis of three RCTs with 107 participants demonstrated accelerated early wound healing and a favorable short-term safety profile, with no serious treatment-related adverse events [51]. Additionally, a pilot trial combining PBMT with resistance exercise in patients with peripheral artery disease and DFU showed improved wound healing and functional mobility, although sample size was small [52]. In a randomized clinical trial, Mirpour *et al.* similarly demonstrated faster wound healing in patients treated with CAP compared with standard care, without significant adverse effects [53]. These findings suggest that CAP may enhance wound repair through antimicrobial, pro-angiogenic, and immunomodulatory mechanisms, although available data remain limited.

Lastly, electromechanical stimulation, such as extracorporeal shockwave therapy (ESWT) has shown potential in clinical and preclinical settings, promoting angiogenesis, enhancing growth factor release, and reducing inflammation [54]. Table 5 synthesizes these adjunct biophysical therapies, specifically their evidence levels, proposed benefits, limitations, and mechanistic pathways in DFU and CLTI care [50–54].

### Advancements in spinal cord stimulation: using 10-kHz spinal cord stimulation for chronic pain management

Researchers have found that SCS may provide effective pain relief for chronic back and leg pain (CBLP), defined as pain lasting ≥12 weeks [49]. The US Food and Drug Administration has approved SCS for treating chronic pain in patients with diabetic neuropathy, complex regional pain syndrome, and post-spinal surgery pain [55]. Chronic pain is associated with significant negative psychosocial impacts, including strained relationships, reduced productivity, suicidal ideation, and increased mortality [56–58]. Furthermore, the economic burden of chronic pain is substantial, estimated at up to $500 billion annually in the United States and as much as 10% of GDP in some European countries [59, 60].

**Figure 6:**
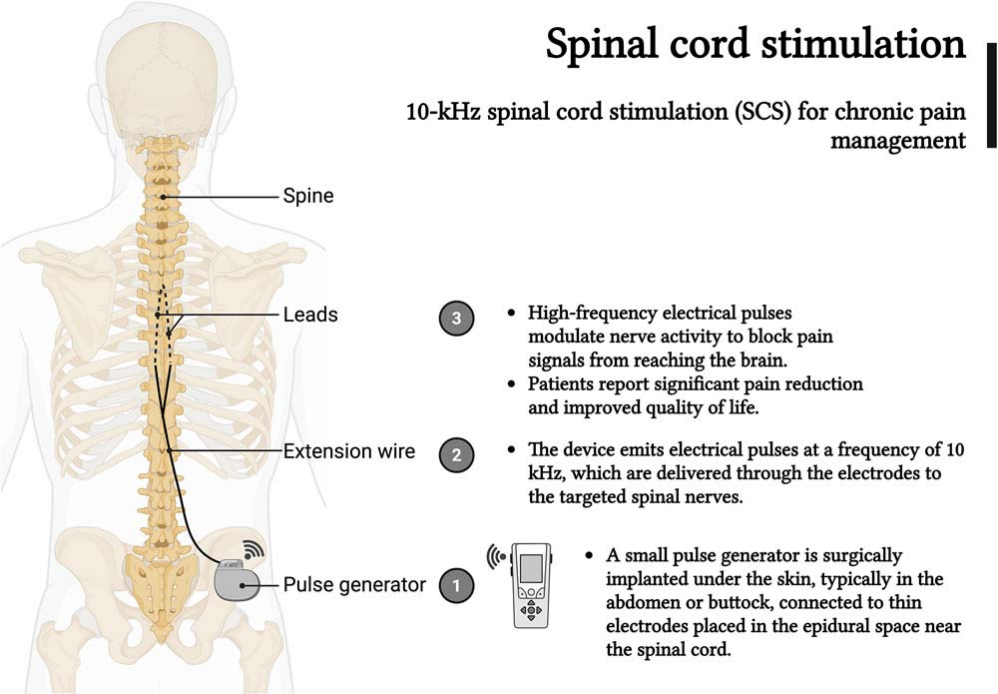
Architecture of a 10-kHz spinal cord stimulation (SCS) system. Epidural leads are positioned adjacent to the dorsal columns of the spinal cord and connected to an implanted pulse generator via extension leads. High-frequency (10-kHz), low-amplitude electrical stimulation modulates neural signaling without producing paresthesia. This neuromodulation may reduce neuropathic pain and influence microvascular perfusion through autonomic and neuroimmune pathways. Created in BioRender. Neal, K. (2026) https://BioRender.com/6okzr41

Traditional treatments include surgery, opioid therapy, medications, and physical therapy [60]. Over the past 40 years, low-frequency SCS (LF-SCS) has been used primarily for failed back surgery syndrome (FBSS) and has demonstrated relative cost-effectiveness [61, 62]. LF-SCS involves surgically implanting electrical leads into the spinal canal to deliver pulses that modulate pain signals (Figure 6) [63]. Despite its efficacy, LF-SCS remains underutilized, in part due to variable provider familiarity, inconsistent payer coverage, and limitations in paresthesia-based targeting [64].

The 10-kHz SCS (Senza® system) developed by Nevro Corporation is a newer technology that provides higher-frequency and lower-amplitude stimulation [65]. It has demonstrated efficacy in treating patients with neuropathic limb pain and those refractory to LF-SCS (Figure 6) [66]. Unlike LF-SCS, 10-kHz stimulation is paresthesia-free, allowing more consistent analgesia across positions and activity levels and potentially increasing patient adherence [67, 68].

Studies have shown promising results for 10-kHz SCS in managing refractory PDN. Petersen *et al.* emphasized the urgent need for improved PDN treatments through a randomized clinical trial evaluating the efficacy and safety of 10-kHz SCS [68, 69]. Petersen et al. emphasized the urgent need for improved PDN treatments through a randomized clinical trial demonstrating the long-term efficacy and safety of 10-kHz SCS, whereas Farber et al. showed that 10-kHz SCS is a cost-effective intervention for patients with refractory PDN [68, 69].

The prospective, multicenter, open-label SENZA-PND RCT enrolled patients with PDN for ≥1 year who were refractory to gabapentinoids and other analgesics. Inclusion criteria included lower limb pain intensity ≥5 cm on a 10-cm visual analog scale (VAS), BMI ≤45, HbA1c ≤10%, and daily morphine equivalents ≤120 mg [28]. Participants received an implanted device delivering 10-kHz SCS alongside conventional medical management (CMM) [28].

The primary endpoint was the proportion of patients achieving ≥50% pain relief on the VAS without neurological worsening at 3 months [28, 70]. Secondary endpoints included changes in pain VAS, neurological exams, quality of life measured by the EuroQol Five Dimension questionnaire, and HbA1c over 6 months [28].

Among 216 randomized patients (mean age 60.8 years), 79% of those receiving 10-kHz SCS plus CMM met the primary endpoint compared to 5% in the CMM-only group, demonstrating a substantial treatment effect [28]. Device-related infections necessitating removal occurred in ~2% of the 10-kHz SCS group [28]. Pain VAS scores decreased significantly in the 10-kHz SCS group versus CMM alone (1.7 cm vs. 6.9 cm) [28]. Neurological improvements were observed in 62% of the 10-kHz SCS group compared to 3% in controls at 6 months [28]. Interestingly, trial stimulation also resulted in improved sensation, potentially due to increased peripheral blood flow, enhanced central sensory processing, or increased intraepidermal nerve fiber density [69, 71].

This study indicates that 10-kHz SCS provides significant pain relief, neurological improvement, and quality-of-life benefits for patients with refractory PDN [69–71]. However, longer-term durability, cost-effectiveness, and comparative effectiveness against other neuromodulation modalities remain an important area of further study.

### Neuromodulation focus on chronic limb-threatening ischemia

Treating chronic back pain can be challenging for some patients, especially when surgical options are limited. In May 2023, Abbott announced US FDA approval of an SCS indication for nonsurgical refractory back pain; the DISTINCT RCT evaluated BurstDR stimulation versus conventional medical management in this population [72]. This trial aimed to evaluate the efficacy of BurstDR dorsal column stimulation compared with conventional standard care in patients with refractory axial lower back pain featuring a neuropathic component, measuring pain improvement over six months [72, 73].

BurstDR therapy delivers bursts of electrical energy without producing paresthesia (tingling sensations), modulating the brain’s interpretation of pain signals. Clinical studies have demonstrated its efficacy, and Abbott’s review indicates that BurstDR is better tolerated by patients (70.8%) compared to traditional tonic stimulation [74, 75]. To date, most comparative studies suggest that BurstDR provides improvement in patient comfort and reductions in paresthesia-related discontinuation, although long-term durability remains under investigation [76].

In the initial phase involving 270 patients with an average back pain duration of 12.8 years, 85.2% of those receiving SCS experienced significant pain reduction, compared with 7.1% in the control group [76]. Over 91% of SCS recipients reported notable pain relief and improved quality of life, with an average pain reduction nearing 70% [77]. Interpretation must consider that this early phase was open label, which may inflate perceived treatment effects; thus, longer blinded follow-up is needed. Regardless, this neuromodulation approach represents a potentially groundbreaking treatment for patients unable to undergo surgery or considered too frail for operative interventions and may complement emerging limb-salvage strategies in CLTI.

### The use of ultrasound-driven splenic stimulation: a non-invasive approach

pFUS stimulation offers a novel approach to neuromodulation by targeting peripheral nerves, neural endings, and organs directly. Early studies in the 1950s on transcranial focused ultrasound (tFUS) demonstrated reversible inhibition of central nervous system function in animal models, particularly within deep brain tissue [78]. However, pFUS remains under investigation due to limited understanding of its exact mechanism, especially its non-thermal effects, and a scarcity of human trials, despite its potential to modulate peripheral neuroimmune circuits.

Preclinical studies have shown that pFUS can modulate the splenic cholinergic anti-inflammatory pathway, reducing cytokine markers such as TNF, IL-1β, and IL-6 in renal ischemia–reperfusion injury models in mice [29, 79]. This was first demonstrated by Cotero *et al.* via implanted vagus nerve stimulation in animals [29, 80]. Expanding on this, hepatic pFUS has been explored for its potential to regulate blood glucose levels and suppress hyperglycemia [29, 81, 82]. These findings support the hypothesis that pFUS can regulate autonomic-immune reflexes with systemic metabolic and inflammatory effects.

Furthermore, Raihane *et al.* reported that non-invasive splenic pFUS stimulation could represent a novel therapeutic approach for burn wound care by improving cytokine profiles through splenic modulation [83]. Preclinical data provided by General Electric on rat models of diabetic ulcers and burn wounds demonstrated significant benefits. In the diabetic ulcer model, a 16-day course of 3-minute daily ultrasound stimulation resulted in notable cytokine changes and accelerated wound healing, with a 75% reduction in wound size achieved 4 days faster than placebo [83]. Similarly, the burn wound model showed accelerated closure-up to 13 days earlier than controls, suggesting a reproducible anti-inflammatory mechanism across injury types [83].

These findings were supported by two clinical trials (NCT04701489 and NCT03548116) utilizing LOGIQ E9 ultrasound systems for precise splenic targeting [83, 84]. Although early-phase, these studies primarily address feasibility and procedural safety, laying groundwork for later efficacy-focused trials.

**Figure 7:**
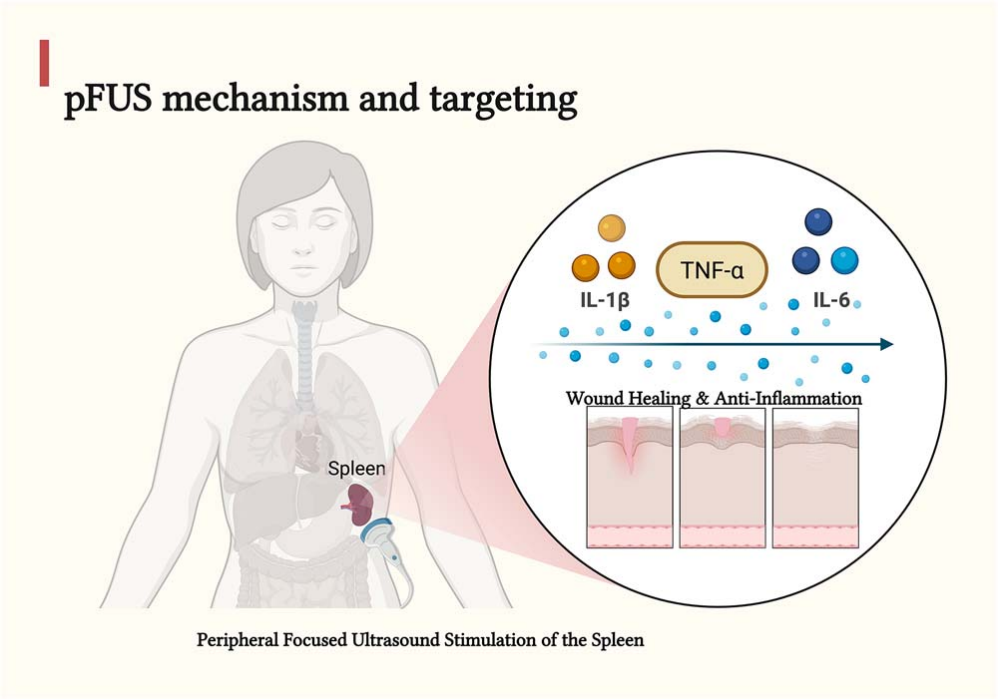
Proposed mechanism of splenic peripheral focused ultrasound (pFUS) stimulation in immune modulation. pFUS stimulation targeting the spleen activates the splenic cholinergic anti-inflammatory pathway, a vagus nerve–mediated neuroimmune reflex. This stimulation reduces pro-inflammatory cytokines including tumor necrosis factor alpha (TNF-α), interleukin-1 beta (IL-1β), interleukin-6 (IL-6), and may enhance nitric oxide signaling. These systemic effects are associated with improved inflammatory balance and accelerated wound healing in preclinical and early-phase clinical studies. Created in BioRender. Neal, K. (2026) https://BioRender.com/rgrubjl

Building on promising preclinical findings, Armstrong *et al.* conducted a pilot clinical trial to evaluate whether pFUS targeting the spleen can accelerate burn wound healing in humans (Figure 7). The study enrolled 24 participants, randomized equally to receive either pFUS treatment or placebo [83–85]. The primary objective is to assess whether stimulation of the splenic anti-inflammatory pathway reduces immune responses and pain, and improves quality of life in patients with partial-thickness burns [85]. Secondary endpoints include time to wound closure, need for debridement, and changes in systemic inflammatory biomarkers, aligning mechanistically with preclinical data [85].

This year-long trial is being conducted at the LA General Regional Burn Center and holds potential not only for improving outcomes in burn wounds but also for broader applications, including diabetic wounds and chronic limb ischemia, and other ischemic inflammatory conditions in which splenic immune modulation may accelerate tissue repair [85]. At the time of manuscript preparation, no peer-reviewed outcomes from this study had been published [85].

### Exploring the potential: proof of concept for remote ischemic conditioning in diabetic foot ulcers

**Figure 8:**
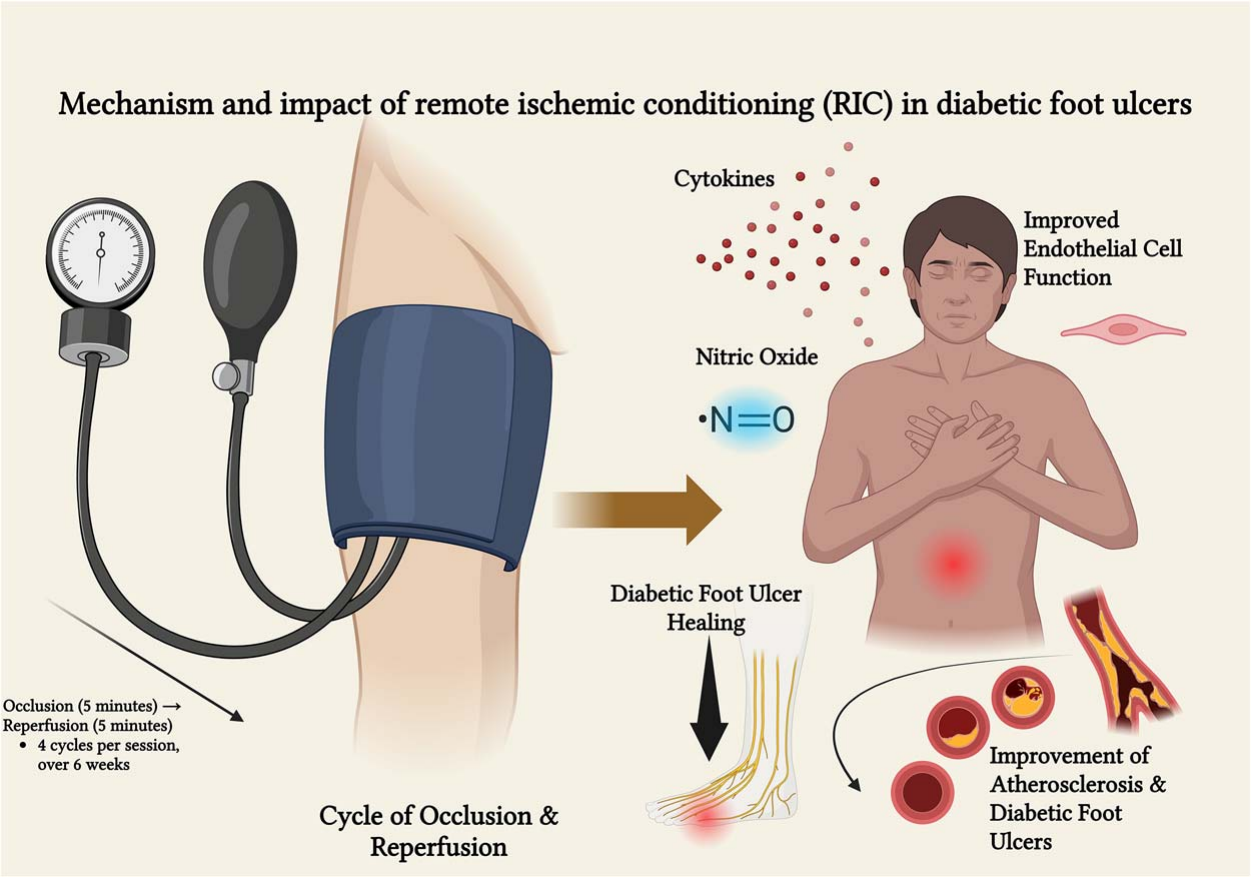
Mechanism of remote ischemic conditioning (RIC) in diabetic wound repair. RIC involves repeated cycles of transient limb ischemia and reperfusion, typically four cycles of five-minute occlusion followed by five-minute reperfusion. This stimulus induces systemic release of circulating protective mediators, including nitric oxide, cytokines, and growth factors. Resulting humoral and endothelial responses improve microvascular function, promote angiogenesis, and may enhance healing of distal ischemic wounds while providing broader vascular protective effects. Created in BioRender. Neal, K. (2026) https://BioRender.com/4nu6bi8

RIC is an emerging, non-invasive treatment showing promise for DFUs. Typically, RIC involves four cycles of alternating 5-minute occlusion and reperfusion, repeated over 6 weeks [23]. This process activates humoral and neural pathways, upregulating anti-inflammatory cytokines, growth factors, and NO, a key mediator of wound healing (Figure 8) [23, 86]. Further, RIC has been shown to influence endothelial function and microvascular perfusion, mechanisms particularly relevant to ischemic diabetic tissue [86].

In a double-blinded RCT by Shaked *et al.*, 34 participants received three RIC treatments spaced every 2 weeks over 6 weeks alongside standard care [24]. The treatment group exhibited a 41% complete healing rate versus 0% in the placebo group. Moreover, 64% of treated subjects achieved a 75% reduction in wound area compared to 25% in controls [24, 85]. A clinical trial is registered to test the effects of RIC on DFUs, measured wound perfusion, biomarkers (VEGF, SDF-1α), and ulcer size [24]. However, published RCT evidence remains limited to small studies, and larger, multicenter trials are needed to confirm durability and generalizability [87, 88]. In preclinical models, RIC has demonstrated vascular protection in diabetes: for instance, in a T1DM rat model, RIC slowed blood-retinal barrier damage [89].

Building on these findings, Regulski *et al.* conducted a proof-of-concept study with 40 subjects (41 wounds) who received RIC three times weekly with standard care for 12 weeks [23]. Subjects not fully healed by 12 weeks but with significant wound reduction were allowed an 8-week extension [23]. By week 20, 75.6% of wounds in the RIC group had healed, compared to 36.6% in the standard care group [23]. For wounds that fully healed in the RIC group, mean wound size reduction was 54.3% [23]. These results align with earlier findings from Shaked *et al.*, demonstrating consistent benefit across studies and reinforcing the plausibility of RIC as a regenerative adjunct for chronic DFUs (Figure 8) [23, 24].

Boghossian *et al.* further explored renal ischemic conditioning using blood pressure cuffs to induce brief limb ischemia (3–5 minutes), stimulating protective systemic responses [35]. They highlighted a gap in the literature: while RIC is well studied in cardiac contexts, research on DFUs is sparse. Their review identified 21 relevant articles, with only six focused on diabetic patients and one specifically on DFUs [90]. This highlights a significant evidence gap and a need for clinical trials tailored to diabetic pathophysiology rather than extrapolated from cardiovascular research [23].

Renal ischemic conditioning traces back to early experimental work describing ischemic preconditioning via cycles of non-lethal ischemia and reperfusion [90, 91]. Later animal studies revealed RIC’s temperature sensitivity and cardioprotective effects from restricted blood flow to lower limb muscles [92, 93]. Additionally, RIC modulates immune responses at transcriptional and post-transcriptional levels [94]. Emerging data suggest that these molecular pathways, particularly shifts in inflammatory gene expression and mitochondrial resilience, may translate to improved healing in peripheral ischemic tissues [95].

Experimental models demonstrate that RIC reduces proinflammatory markers and protects multiple organs, including muscle flaps, lungs, liver, brain, and heart, from ischemia–reperfusion injury [96]. Studies also suggest improved endothelial function, tissue oxygenation, and benefits in DFUs and atherosclerosis [95, 96]. Despite these promising findings, further research is needed to confirm clinical efficacy.

To bridge this gap, Boghossian *et al.* proposed replicating RIC in human volunteers using standard blood pressure cuffs to induce supra-systolic ischemia–reperfusion cycles (3–5 min), based on preclinical and theoretic work [35]. If proven effective, this approach could support RIC as a cost-effective method to reduce morbidity in DFU patients [35]. Such accessibility makes RIC particularly compelling for resource-limited settings, where DFU rates are high and advanced wound care options may be limited.

An emerging, hypothesis-generating framework is that multiple “physiologic action at a distance” therapies may converge on autonomic reflex circuitry, including vagus nerve–mediated neuroimmune pathways that regulate systemic inflammation and endothelial function. The clearest evidence for this concept comes from splenic-targeted pFUS stimulation, which has been shown to engage the splenic cholinergic anti-inflammatory pathway, a classically vagus-dependent circuit associated with reduced pro-inflammatory cytokine signaling [20, 80]. RIC, although traditionally characterized by circulating humoral mediators, also demonstrates increasing support for neural and autonomic contributions within its protective cascade [44, 90]. SCS, in contrast, exerts more direct autonomic effects through sympathetic modulation and subsequent improvements in ischemic perfusion [31, 46]. Considered together, these observations suggest that autonomic signaling may serve as a shared mechanistic substrate across otherwise distinct modalities, a proposition that warrants direct mechanistic validation in future studies [20, 44, 80, 90].

### Use of tibial transverse transport for critical limb-threatening ischemia

Another study examined neuromodulation strategies for diabetic foot, a severe diabetes complication associated with high mortality. Treatment traditionally focuses on blood glucose control and wound management. While effective for Wagner grade 1–2 ulcers, outcomes for grade 3 and above have been less favorable [97, 98]. Consequently, TTT was developed and has been increasingly reported as a limb-salvage strategy for severe diabetic foot cases. TTT was originally adapted from the Ilizarov technique and is increasingly applied in patients who are poor candidates for conventional revascularization [98, 99].

TTT has demonstrated significant improvements in distal limb blood circulation in patients with lower limb ischemic disease. Clinical researchers have also noted improvements in skin sensation, temperature, and wound healing since 2019 [99]. These clinical findings are thought to arise from TTT-induced mechanical stimulation of the periosteum, which promotes angiogenesis, enhances microcirculatory flow, and may exert neuromodulatory effects through peripheral nerve regeneration pathways [100].

These changes were monitored using high-frequency color Doppler ultrasonography (HFCDU), which tracks blood flow and distinguishes arterial from non-arterial stenosis. HFCDU was specifically selected due to its sensitivity in detecting dynamic postoperative perfusion changes [97].

Liao *et al.* enrolled 25 patients (20 male, 5 female), aged 51–78 years, with Wagner grade 3–5 diabetic foot, treated with TTT at Renmin Hospital of Wuhan University between January 2021 and August 2022 [97]. Inclusion criteria included age 18–80 years and willingness to undergo TTT; exclusion criteria included ≥75% stenosis of the popliteal artery (POA) on the affected side, anesthesia contraindications, or recent cardio-cerebrovascular events [97]. This study design ensured that outcomes were evaluated in a population not amenable to standard revascularization, increasing relevance to real world CLTI management.

Ultrasonography (Philips EPIQ5) assessed arteries including the CFA, SFA, POA, ATA, and dorsalis pedis. Measured parameters included inner diameter, intima-media thickness, peak flow velocity, resistance index, blood flow, atherosclerotic plaques, and medial arterial calcification [97]. Vascular assessment allowed investigators to differentiate TTT-related microvascular improvements from changes driven by large-vessel disease progressions [97].

The TTT procedure was performed under nerve block and/or general anesthesia. A 10-cm arc incision was made medially on the tibia to create a bone window for tibial transport. External fixation screws and lateral tibial transport frames were installed. Thorough debridement of ulcers, damaged bone, blackened toes, and necrotic tissue was performed. Wound dressing and bone transport commenced one week postoperatively. This protocol aligns with contemporary orthopedic standards for minimizing infection risk and optimizing early angiogenic responses [97].

Results showed significantly shorter wound healing times in the non-arterial stenosis group compared to the arterial stenosis group, though both groups achieved 100% healing within the reported follow-up period [97]. The non-arterial stenosis group had lower extremity arteriopathy scores and higher preoperative POA blood flow. One-month post-op, POA flow decreased in the non-arterial group but increased significantly in the arterial stenosis group, though still lower than the non-arterial group. Plantar microcirculation was initially better in the non-arterial group but improved significantly in the arterial stenosis group by one month [101]. These results suggest a differential response to TTT based on preexisting vascular pathology, supporting microcirculatory, rather than large-vessel, mechanisms as the primary drivers of clinical improvements [97, 101].

Both animal and clinical studies showed new blood vessel formation around transported bone blocks during TTT, creating collateral circulation. Computed tomography angiography (CTA) and perfusion imaging revealed angiogenesis and increased blood flow three months post-TTT. This growing body of evidence supports TTT as a combined mechanical and neuromodulatory therapy that enhances skin, vascular, and peripheral nerve regeneration in diabetic foot patients [101–103].

### Periosteal distraction and lateral tibial periosteum distraction as limb-salvage strategies

In a case report, Wang *et al.* described LTPD, a novel and less invasive technique than TTT for foot preservation in CLTI and non-healing ulcers after failed vascular interventions [45]. LTPD uses controlled periosteal tension to stimulate angiogenesis without requiring cortical bone cutting, thereby reducing procedural morbidity [43, 104, 105]. LTPD further improves pain relief, limb function, and wound healing by promoting microcirculation via mechanical stimulation and vascular network regeneration [43, 104, 105].

The patient, a 70-year-old male smoker with CLTI and a non-healing ulcer, had previously undergone stenting and angioplasty with minimal benefit [45]. Due to poor vascular status, LTPD was performed as a limb salvage strategy. Postoperatively, the patient experienced significant pain relief, improved skin temperature, and restored independent mobility. At three months, no symptom recurrence was noted; ankle-brachial index (ABI) improved from 0.5 to 0.9, and visual analog pain score decreased from 9 to 1. While limited to a single case, these findings show LTPD’s potential role for patients who have exhausted conventional revascularization pathways [45].

Wang *et al.* also reviewed 42 articles on PD osteogenesis and angiogenesis, grounding their analysis in Ilizarov’s law of tension-stress, which describes mechanical tension stimulating tissue growth and regeneration [45, 106]. Their synthesis emphasizes that periosteal based therapies may offer a biological workaround in CLTI cases where endovascular options are unsuccessful or unavailable [106]. While focused on CLTI, their findings suggest PD techniques may be applied to DFU treatment as a limb-salvage option. Although mostly studied in animals, further human research is warranted [106, 107].

PD osteogenesis relies on distraction histogenesis, where gradual periosteal stretching induces robust vascular ingrowth and tissue regeneration [108]. Its similarity to TTT in promoting angiogenesis, combined with potentially reduced invasiveness, positions it as an emerging therapeutic modality requiring standardized protocols and controlled trials before widespread adoption [108–110]. The current clinical evidence supporting TTT and LTPD is derived largely from single-center observational cohorts, retrospective analyses, and case reports, predominantly from a limited number of geographic regions. These study designs introduce substantial risks of selection bias, publication bias, and overestimation of treatment effect, particularly in the absence of blinded outcome assessment or standardized comparators. Firm conclusions regarding comparative effectiveness, durability of benefit, and generalizability across broader diabetic foot and CLTI populations cannot yet be drawn. Larger, multicenter RCTs with standardized endpoints are required before these techniques can be routinely recommended outside specialized centers.

### Surgical wound treatment: a combination of tibial transverse transport, skin grafting techniques, debridement, and vacuum sealing drainage

Chang *et al.* conducted a study in 2021 involving 13 diabetic foot patients with Wagner grade 3–4 injuries treated using ISWT for severe chronic limb ischemia [111]. ISWT combines TTT, debridement, vacuum sealing drainage (VSD), and skin grafting. Blood glucose levels were controlled preoperatively [111]. Outcome measures included wound healing time, skin temperature at the dorsum midpoint of the affected foot, VAS pain scores, and ABI. Assessments were performed before and after surgery, including CTA of the lower extremity arteries to evaluate small arteriolar formation and detect complications [111]. This multimodal approach aims to address both macro- and microvascular dysfunction, a key challenge in diabetic foot pathology.

Among patients aged 45–66 years with 3–13 months of follow-up, all achieved full wound healing without amputation. One complication was reported: a nail channel infection. The mean healing time was 25.8 ± 7.8 days (range 17–39 days), with external fixation scaffolds worn for an average of 71.8 ± 10 days (range 56–91 days). Patients resumed walking after a mean of 30.8 ± 9.1 days (range 18–45 days). Significant improvements were noted in skin temperature, VAS scores, and ABI within 3 months postoperatively. CTA showed increased numbers of lower extremity arteries, thickened small arterioles, and collateral circulation formation [112].

These radiologic and clinical findings further support the hypothesis that TTT based approaches stimulate angiogenesis and microvascular remodeling, contributing to improved perfusion and tissue repair [97, 111, 113].

These promising findings are relevant because DFUs often result from diabetes-induced microcirculation disorders and ischemia. However, the authors note that the reliance on external fixation scaffolds present a meaningful barrier to patient comfort and mobility, potentially limiting widespread adoption [111]. Additionally, the small sample size and relatively short follow-up highlight the need for larger studies assessing long-term tissue quality and the role of ISWT in chronic limb ischemia treatment. Long-term durability of neovascularization and recurrence risk remain unclear [113, 114].

Separately, a recent rat study explored the role of calcium ion recruitment in bone healing during TTT. Using double fluorescent staining and laser capture microdissection (LCM), researchers examined expression of calcium channels in the Orai1/STIM1 pathway around bone tissue [111]. OPN was identified as a key protein, shown in vitro to enhance angiogenesis of human umbilical vein endothelial cells (HUVECs) and activate the Orai1/STIM1 pathway [36, 111]. Activation of this pathway increased endothelial nitric oxide synthase (eNOS) expression, promoting NO release and accelerating wound healing. In diabetic mouse models, OPN stimulation facilitated skin wound repair, suggesting a promising, non-invasive TTT-based approach to improve diabetic wound healing and patient quality of life [36, 111]. These preclinical findings provide a biologic foundation for TTT’s regenerative effects and point toward adjunctive molecular targets that may enhance future limb-salvage therapies.

### Implementation challenges: cost, access, safety, regulation, and research priorities

While early efficacy data for regenerative and neuromodulation therapies are encouraging, several implementation challenges must be addressed before these interventions can be broadly adopted in DFU and CLTI populations. Cost is a major barrier for both researchers and patients. For example, the median device acquisition cost for high-frequency SCS (HF-SCS) is $35 755, with total healthcare utilization decreasing by ~$853 per month during the first six months post-implant [115]. Although Medicare reimburses spinal cord stimulator implantation (CPT 63685), the high device and procedural costs, median HF-SCS acquisition ~$35 755, result in substantial out-of-pocket and system-level expenses, despite early evidence of reduced healthcare utilization post-implant [116, 117]. In economic modeling from 2022, despite large up-front costs, SCS may become cost-saving or neutral after 2–3 years [118].

Accessibility is another concern. Implantable neuromodulation like SCS requires surgical infrastructure, imaging, and postoperative follow-up, which may not be available in low-resource or rural settings. By contrast, non-implant approaches (e.g. pFUS, RIC) may offer lower infrastructure demands, but standardized protocols, dosing regimens, and safety parameters are not yet well established, thus limiting scalability.

Long-term safety data are limited. While short-term explantation rates for HF-SCS are low (e.g. 2.1% at six months in one real-world cohort), long-term device durability, lead migration, infections, and external fixation–related morbidity (for techniques like TTT) remain poorly understood [115]. A recent meta-analysis of permanent SCS implants reported explantation rates of roughly 10%, often due to lead failure, inadequate pain relief, or infection [119]. These risks may be magnified in patients with diabetes, poor wound healing, or peripheral vascular disease.

Other challenges include regulatory and ethical considerations. While SCS is FDA approved for certain pain conditions (with accumulating real-world cost–benefit data), many regenerative modalities, such as pFUS, LTPD, and TTT, remain early-phase or investigational [120, 121]. Translating preclinical findings to first-in-human trials will require rigorous Institutional Review Board oversight, well defined inclusion criteria, and transparent patient consent, as some effects may modulate systemic immune or vascular pathways.

### Risks, failure modes, and long-term prognosis

While neuromodulation and regenerative therapies are promising, they are not without risk and uncertainty. For SCS, lead migration remains a clinically relevant complication, with a recent meta-analysis finding an overall migration rate of 10%, with many cases necessitating lead revision or explanation [122]. Subsequently, subacute postoperative studies report high rates of caudal lead migration, particularly in patients with elevated BMI [123].

Surgical techniques like TTT and external fixator-based bone transport (e.g. Ilizarov) carry their own risks. Pin-tract infection is common; in one cohort, ~44.6% of patients developed such infections [124]. Longer-term external fixation has been associated with major complications including axial deviations, delayed union, and nonunion in retrospective case-series [125]. In another large study of tibial infected non-unions, patients with large bony defects (≥11) had significantly higher complication rates [126].

Other risks include hardware failure, nonunion at the docking site, and the patient burden of prolonged external fixation [127]. External fixation is also associated with prolonged patient discomfort and mobility issues; one prospective study noted earlier conversion to intramedullary nailing reduced fixation time, but this strategy still carries risk of infection recurrence [128].

For pFUS and RIC, the risk profile is less characterized. Limited human data exist on potential off-target effects of repeated ultrasound stimulation, and the long-term sequelae of repeated ischemia in RIC, especially in patients with vascular disease, remain unknown. Long-term outcomes, including amputation-free survival, wound recurrence, and quality of life beyond 12–24 months, have not been systematically assessed for these modalities.

Lastly, a key caveat is that the mechanistic and efficacy data primarily derive from preclinical models, providing biological insights but not establishing clinical effectiveness for SCS, pFUS, RIC, TTT, or LTPD. Translation into diabetic-foot and CLTI populations will require human trials with condition-specific endpoints.

#### Research priorities

- Design multicenter, adequately powered RCTs comparing each modality plus standard wound care versus standard care alone, with primary endpoints such as 12- and 24-month amputation-free survival and ulcer recurrence.
- Harmonize mechanistic and clinical endpoints (e.g. TcPO₂, toe pressure, angiographic perfusion, circulating cytokine panels) to link biology with clinically meaningful outcomes.
- Prioritize pragmatic trials in resource-limited settings for low-cost interventions (RIC), and equity-focused comparative studies to avoid widening disparities.
- Establish prospective safety registries and standardized reporting of device-related complications (lead migration, infection, fixation morbidity) to inform cost-effectiveness and policy maker decisions.

## Conclusions

In conclusion, the escalating prevalence of chronic diseases like obesity and type 2 diabetes highlights the urgent need for innovative, effective, and scalable therapies to reduce the burden of diabetic complications. Emerging neuromodulation technologies such as pFUS, advanced SCS systems, and splenic ultrasound stimulation offer promising non-invasive options for managing diabetic neuropathic pain and enhancing wound healing. Additionally, innovative surgical techniques like TTT and LTPD have shown significant potential in advancing limb salvage, particularly for patients who have exhausted standard revascularization options. RIC further complements these approaches by boosting vascular function in patients who may be ineligible for more invasive interventions. Across these modalities, the shared therapeutic theme is the activation of endogenous regenerative and immunomodulatory pathways, offering a shift from purely palliative wound management toward biologically restorative care. Implementation challenges, including cost, accessibility, long-term safety, and regulatory oversight, remain important considerations for translating these therapies into routine clinical practice. Long-term outcomes, including amputation-free survival, quality of life beyond 12–24 months, and wound recurrence, have not been systematically assessed for most modalities, necessitating the need for careful monitoring of durability and potential late complications. Taken together, these therapies underscore a paradigm shift toward patient-centered regenerative and minimally invasive interventions designed to preserve limbs and improve long-term function. However, despite encouraging early evidence, rigorous multicenter trials, standardized protocols, and long-term follow-up evaluations are needed to validate efficacy, optimize treatment algorithms, and identify patient populations most likely to benefit. With continued research and clinical investment, these novel neuromodulatory and surgical innovations have the potential to reduce amputation rates, improve quality of life, and substantially reduce the global burden of diabetic foot disease.

## Data Availability

Not applicable. No datasets were generated or analyzed for this study.
